# Patterned arrays of lateral heterojunctions within monolayer two-dimensional semiconductors

**DOI:** 10.1038/ncomms8749

**Published:** 2015-07-22

**Authors:** Masoud Mahjouri-Samani, Ming-Wei Lin, Kai Wang, Andrew R. Lupini, Jaekwang Lee, Leonardo Basile, Abdelaziz Boulesbaa, Christopher M. Rouleau, Alexander A. Puretzky, Ilia N. Ivanov, Kai Xiao, Mina Yoon, David B. Geohegan

**Affiliations:** 1 Center for Nanophase Materials Sciences, Oak Ridge National Laboratory, Oak Ridge, Tennessee 37831, USA; 2 Materials Science and Technology Division, Oak Ridge National Laboratory, Oak Ridge, Tennessee 37831, USA; 3 Departamento de Física, Escuela Politécnica Nacional, Quito 170525, Ecuador

## Abstract

The formation of semiconductor heterojunctions and their high-density integration are foundations of modern electronics and optoelectronics. To enable two-dimensional crystalline semiconductors as building blocks in next-generation electronics, developing methods to deterministically form lateral heterojunctions is crucial. Here we demonstrate an approach for the formation of lithographically patterned arrays of lateral semiconducting heterojunctions within a single two-dimensional crystal. Electron beam lithography is used to pattern MoSe_2_ monolayer crystals with SiO_2_, and the exposed locations are selectively and totally converted to MoS_2_ using pulsed laser vaporization of sulfur to form MoSe_2_/MoS_2_ heterojunctions in predefined patterns. The junctions and conversion process are studied by Raman and photoluminescence spectroscopy, atomically resolved scanning transmission electron microscopy and device characterization. This demonstration of lateral heterojunction arrays within a monolayer crystal is an essential step for the integration of two-dimensional semiconductor building blocks with different electronic and optoelectronic properties for high-density, ultrathin devices.

## Abstract (web summary)

Lateral heterojunctions between two-dimensional semiconductor crystals are essential building blocks for electronic devices. Here, the authors utilize electron-beam lithography and selective conversion to simultaneously fabricate arrays of molybdenum diselenide–molybdenum disulfide heterojunctions.

## Graphical abstract

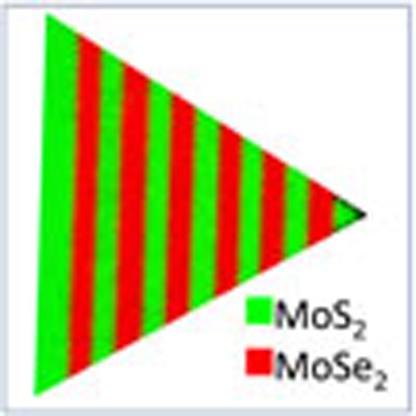

Two-dimensional (2D) layered semiconductors^1^,^2^, notably the transitional metal dichalcogenide (TMD) families (MX_2_: M=Mo, W, Nb; X=S, Se, Te)^1^ in which M atoms are sandwiched between two planes of X atoms, have recently attracted significant attention due to their novel electronic and optoelectronic properties and potential applications for new generations of flexible field-effect transistors^3^,^4^, photovoltaics^5^, light-emitting diodes^6^ and sensors^7^. High-quality mono- and few-layer samples for prototype devices have been synthesized via various techniques including mechanical cleavage^8^, chemical exfoliation^9^,^10^, vapour phase transport^11^,^12^ and laser-based methods^13^,^14^. However, the next challenge for 2D material-based ultrathin integrated circuits is the controllable formation of semiconductor heterojunctions, either by vertically stacking different layers or by formation of lateral heterojunctions of different semiconductors within a single layer^15^,^16^,^17^. Lithographic patterning and compatible synthesis approaches are required for high-density integration of semiconductor heterojunctions, as was recently demonstrated for lateral graphene interconnects embedded within an insulating monolayer of boron nitride by etching and regrowth processes^17^. Alternatively, phase engineering of patterned regions of MoS_2_ crystals by exposure to liquid *n*-butyl lithium has recently achieved the transformation from a semiconducting to a metallic, metastable phase within a single layer^4^.

The most commonly explored method for the formation of a single heterojunction is vapour transport growth (VTG), which has resulted in lateral and vertical epitaxy between semiconducting TMDs such as MoS_2_/MoSe_2_, MoSe_2_/WSe_2_ and MoS_2_/WS_2_ (refs 18, 19, 20). By alternating precursor vapours and adjusting the growth temperature, disparate materials can be nucleated and grown on the edges (lateral junctions) or on top (vertical junctions) of as-grown crystals. However, typical VTG involves well-known challenges of highly non-uniform spatial control over nucleation, growth and layer control, owing to poor understanding and control over gas flow dynamics, chemical reactions and boundary layer diffusion. So far, these challenges as well as contamination by reaction byproducts, and reactivity at exposed crystal edges have limited the scalable and controllable formation of patterned heterojunctions between TMDs by this route.

Here we demonstrate the formation of patterned arrays of lateral heterojunctions between 2D layered semiconductors, MoSe_2_/MoS_2_, within the confines of a single monolayer MoSe_2_ crystal. Heterojunction arrays are formed by sulfur replacement of Se (refs 21, 22) within MoSe_2_ (lattice constant 3.288 Å, optical bandgap 1.55 eV)^19^,^23^, converting it to MoS_2_ (lattice constant 3.160 Å, optical bandgap 1.85 eV)^24^,^25^ in predefined locations. Using well-developed and scalable photolithography and electron beam lithography processes, a variety of patterns exposed on monolayer MoSe_2_ crystals are selectively converted to MoS_2_ by pulsed laser vaporization of sulfur, producing predefined arrays of lateral MoSe_2_/MoS_2_ heterojunctions within a single monolayer. This process provides a unique capability for the digital delivery of a precise amount of sulfur atoms with super-thermal kinetic energies during the conversion process, allowing controllable sulfurization for either alloying or total conversion of MoSe_2_ to MoS_2_. Raman and photoluminescence (PL) spectroscopy and mapping confirm the formation of periodic heterojunctions. High-angle annular dark-field atomic resolution scanning transmission electron microscopy (STEM) reveals that the MoS_2_ is comparable in quality to pristine material, with sharp (∼5 nm) heterojunction boundaries. This controllable and versatile formation of lithographically patterned lateral heterojunction arrays offers the potential for their integration as 2D layer ‘building blocks', along with metal/insulator domains, for next-generation electronics.

## Results

### Formation of patterned arrays of lateral heterojunctions

**Figure 1:**
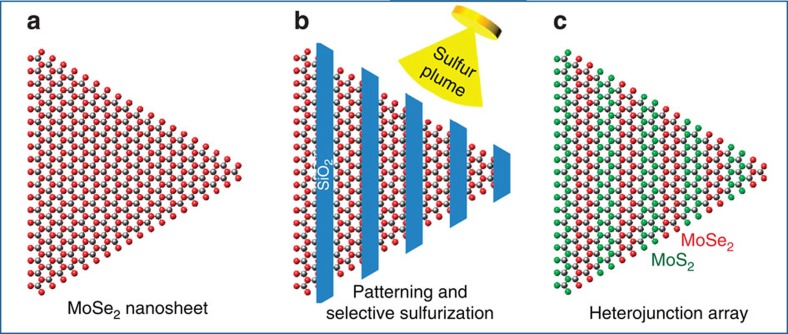
Schematic illustration of the experimental steps for the formation of MoSe_2_/MoS_2_ heterojunction arrays. (**a**) Starting MoSe_2_ monolayer crystal. (**b**) MoSe_2_ is patterned by e-beam lithography and SiO_2_ deposition, followed by sulfurization of uncovered areas. The SiO_2_ mask is used to prevent the underlying MoSe_2_ regions from reacting with sulfur while the exposed regions are converted to MoS_2_. (**c**) Formation of arrays of lateral MoSe_2_/MoS_2_ heterojunctions within the monolayer crystal.

Figure 1 schematically illustrates the experimental steps for the formation of periodic lateral heterojunctions. The starting monolayer MoSe_2_ crystals with lateral sizes ranging from 10 to 100 μm were synthesized using a previously reported method^11^,^13^ (Supplementary Fig. 1; Supplementary Note 1). The crystals were then simply masked by conventional patterning processes, followed by selective conversion of the unmasked MoSe_2_ to MoS_2_ by pulsed laser vaporization of sulfur. The masking material (∼50 nm SiO_2_) was electron beam evaporated and was found to be quite effective in protecting the areas of the 2D crystal underneath from sulfurization.

Prior to the patterning process and formation of heterojunction arrays, the conversion of 2D monolayers was studied at various temperatures for different numbers of sulfur pulses to understand and optimize the process (Supplementary Note 2). PL and Raman spectroscopies were used to investigate the crystal conversion process at various conditions and to reveal their optical properties (Supplementary Fig. 2). We found that MoSe_2_ nanosheets are totally converted to MoS_2_ for substrate temperatures above 600 °C and 300 laser-vaporized sulfur pulses, whereas temperatures and laser pulses below 600 °C and 300 pulses, respectively, resulted in the formation of intermediate compositions. At temperatures above 800 °C, decomposition and damage of the crystals occurred. Therefore, substrate temperatures were fixed at 700 °C with 400 sulfur pulses to ensure a complete conversion of MoSe_2_ to MoS_2_. Conversion of WSe_2_ to WS_2_ was also achieved by the same process (Supplementary Fig. 3; Supplementary Note 3) to demonstrate the broad applicability and advantages of the technique.

The thermodynamic stability and optical bandgaps of various 2D transition metal dichalcogenide alloys (MX_2(1−*x*)_X′_2*x*_ where M=Mo, W and X, X′=S, Se, Te) have previously been computationally predicted to understand the mixing and phase-segregation behaviour for different alloy compositions^26^,^27^. Of these, MoS_2*x*_Se_2(1−*x*)_ alloys were found to have the lowest free energy of mixing^26^,^27^, yet all MX_2(1−*x*)_X′_2*x*_ were predicted to form stable alloys with complete miscibility at the moderate temperatures typically employed during chemical vapour deposition or bulk synthesis, and with continuously tunable direct bandgaps, making them good candidates for 2D optoelectronics^26^.

### Spectroscopic characteristics of heterojunctions

**Figure 2:**
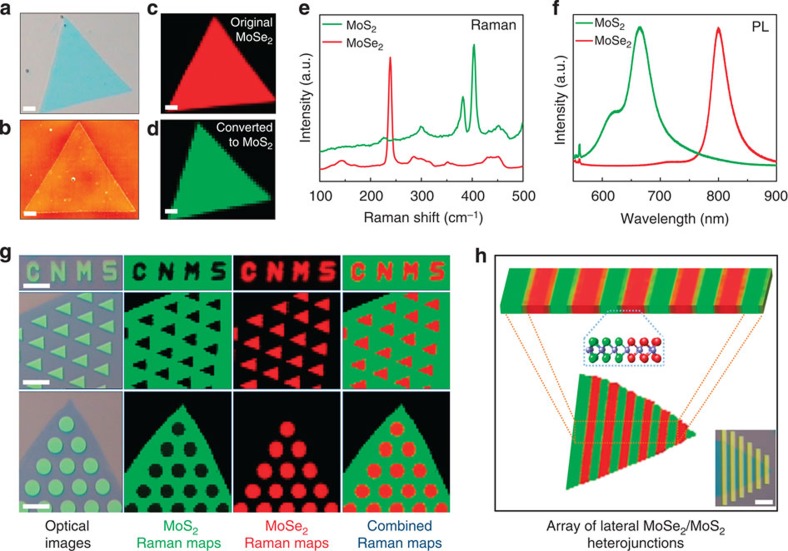
Conversion of MoSe_2_ to MoS_2_ and the formation of lateral heterojunction arrays. (**a**,**b**) Optical and atomic force microscopy images of a typical MoSe_2_ monolayer with a lateral size of ∼40 μm. (**c**,**d**) Raman maps of a monolayer nanosheet before and after the complete conversion process (400 pulses at 700 °C), respectively, indicating uniform intensity across the entire crystal. (**e**,**f**) Representative Raman and PL spectra of the pristine MoSe_2_ and converted MoS_2_ regions. (**g**,**h**) Various examples of lateral heterojunction arrays formed within monolayer crystals by patterning and selective conversion processes. The green, red and combined Raman maps are obtained from corresponding optical images, representing the MoS_2_ (intensity map at 403 cm^−1^), MoSe_2_ (intensity map at 238 cm^−1^) and overlaid MoSe_2_/MoS_2_ regions, respectively. Scale bars, 5 μm.

Raman and PL spectroscopy were used to probe the structures spatially, monitor the conversion degree and to map the heterojunction arrays within the monolayer crystals. Figure 2a,b shows optical and atomic force microscopy images of a typical MoSe_2_ 2D crystal. The corresponding Raman maps of the crystal before and after the full conversion process are shown in Fig. 2c,d. The MoSe_2_ and MoS_2_ Raman maps are plotted for the E^1^_2g_ mode of MoSe_2_ at 238 cm^−1^ and E^1^_2g_ modes of MoS_2_ at 403 cm^−1^, respectively^10^,^13^. Representative Raman and PL spectra of the flake before and after the conversion process are shown in Fig. 2e,f, indicating MoSe_2_ and MoS_2_ Raman peaks similar to those reported in the literature^10^,^13^. The uniform intensity distribution in the Raman maps indicates spatial uniformity of the 2D crystals both before and after the conversion. Figure 2g,h shows the formation of various lateral heterojunction arrays prepared by our patterning and selective conversion process. Similarly, the uniform intensity of Raman maps clearly indicates the spatial uniformity of the MoSe_2_ and MoS_2_ domains, and formation of heterojunction arrays within the monolayer crystals. The Raman and PL spectra obtained from the pristine and converted regions of the crystals are similar to the ones shown in Fig. 2e,f.

### STEM characterization of heterojunctions

**Figure 3:**
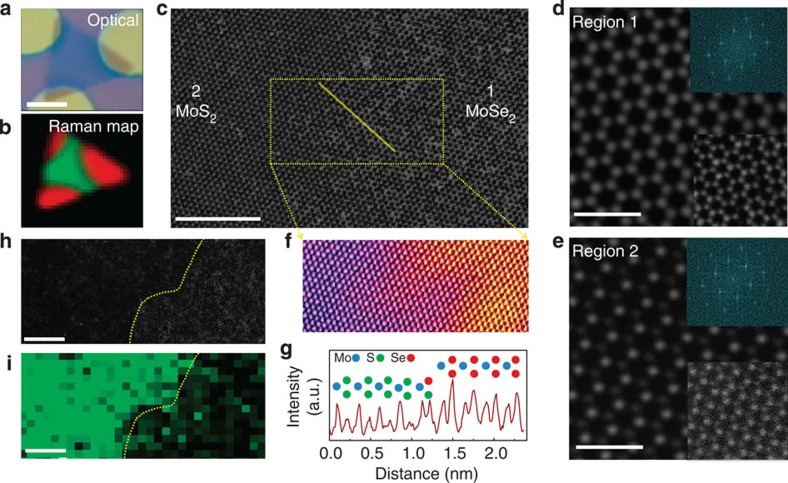
STEM Z-contrast image and elemental imaging of a heterojunction. (**a**,**b**) Optical image and corresponding Raman map of a patterned nanosheet on a SiO_2_ substrate (scale bar, 5 μm). The SiO_2_ masks (circular discs in **a**) are removed during KOH etching, and the transfer of the nanosheet onto the TEM grids. (**c**) Low-magnification Z-contrast image of the nanosheet showing the MoSe_2_ and MoS_2_ regions with a finite boundary across the domains (scale bars, 5 nm). (**d**,**e**) Fourier filtered images of the atomic resolution Z-contrast images of the MoSe_2_ and MoS_2_ (bottom insets in the images) domains with corresponding fast Fourier transform patterns (top insets in the images). (**f**,**g**) Surface and line intensity profiles of the squared and line-marked regions in **c**. (**h**,**i**) Low-magnification image of a boundary with its corresponding electron energy loss spectroscopy map showing the sulfur concentration (scale bars, 5 nm).

Crystalline structures of the converted and pristine regions as well as their heterojunction boundaries were also studied by atomic resolution Z-contrast STEM. Figure 3a,b shows the optical image and corresponding Raman map of a typical patterned/converted layer transferred onto a grid for STEM imaging (Supplementary Fig. 4; Supplementary Note 4). As shown in Fig. 3c, atomic resolution Z-contrast STEM images are taken at the heterojunction that clearly shows both MoSe_2_ (Fig. 3d) and MoS_2_ (Fig. 3e) 2D crystal domains. The line and surface intensity profiles of the selected regions in Fig. 3c are shown in Fig. 3f,g. Likewise, Fig. 3h,i shows a STEM image of a boundary with its corresponding electron energy loss spectroscopy map showing the sulfur content (in green) as a function of position. It is clear that both pristine MoSe_2_ and converted MoS_2_ regions lie within the same honeycomb lattice with no grain boundaries—that is, the MoSe_2_ crystal serves as a template, maintaining the same crystal orientations throughout the whole structure. As can be seen from the STEM image, the interface has a finite width similar to that reported for MoSe_2_/WSe_2_ heterojunctions^19^. The boundary appears to be a MoS_*x*_Se_1−*x*_ ternary alloy with a composition gradient over a distance of several nanometres. The sharpness of the heterojunctions is related to the e-beam lithography and patterning processes used in this work, which can be improved further.

### Electrical transport properties

**Figure 4:**
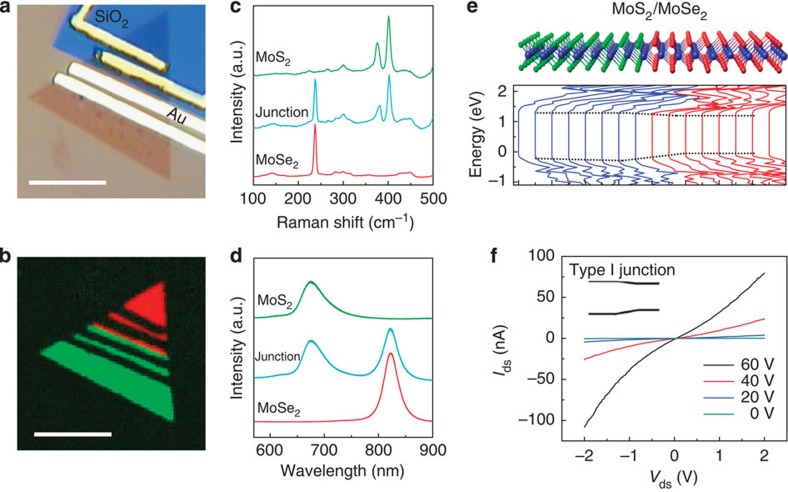
Electrical transport properties. (**a**,**b**) Optical and Raman map of a MoS_2_/MoSe_2_ (green/red) heterojunction device. (**c**,**d**) Raman and PL spectra of MoS_2_ and MoSe_2_ obtained from each region showing a distinct peak for regions away from the junction and appearance of both peaks at the junction. (**e**) First-principles DFT calculation of MoSe_2_ and MoS_2_ superlattices showing the formation of type-I band alignment. (**f**) *I*_ds_*−V*_ds_ characteristics of the heterojunction showing an n–n junction behaviour at the interface.

The electrical transport properties of pristine MoSe_2_, converted MoS_2_ and their heterojunctions were studied by forming a device structure. Figure 4a shows an example of a device structure fabricated in this work. Prior to the conversion process, two electrodes were fabricated on one side of the crystal to measure the transport properties of the pristine MoSe_2_. Then, this part was covered with a SiO_2_ mask and the sulfurization was carried out, resulting in the conversion of the exposed half of the crystal to MoS_2_. Two electrodes were then fabricated on this half to measure the electrical transport characteristics of the converted MoS_2_ region. Figure 4b shows the Raman map of the device with red and green colours clearly indicating the MoSe_2_ and MoS_2_ regions, respectively, and their boundaries. Representative Raman and PL spectra of these regions are also shown in Fig. 4c,d, indicating distinct Raman and PL peaks for each domain while observing simultaneous MoSe_2_ and MoS_2_ peaks right at the junction.

To understand band alignment at the interface, first-principles calculations, based on density functional theory, were performed using the Vienna *ab initio* Simulation Package (Supplementary Note 5). As illustrated in Fig. 4e, we considered a supercell in which eight zigzag rows of MoS_2_ joined eight rows of MoSe_2_, and plotted the partial density of states projected onto the atomic rows in the MoS_2_ and MoSe_2_ across the heterojunction. From the projected density of states, we can see that there exists an obvious graded bandgap across the heterojunction. In particular, we find that the lateral MoS_2_/MoSe_2_ heterojunction forms a type-I band alignment, as indicated by the dotted line (Fig. 4e), since the bandgap of MoS_2_ entirely overlaps that of MoSe_2_.

*I*_ds_*−V*_ds_ curves were obtained from pristine MoSe_2_, converted MoS_2_ and their heterojunction to investigate their transport characteristics. According to the measured *I*_ds_*−V*_ds_ curves, both MoSe_2_ and converted MoS_2_ show n-type behaviour (Supplementary Fig. 5) similar to the previously reported work^11^,^12^. Consequently, as shown in Fig. 4f, their boundary shows transport characteristic of an n–n heterojunction. The smaller current amplitude across the heterojunction boundary may be attributed to electron scattering at the heterojunction and the type of their band alignment.

## Discussion

In conclusion, through lithographic patterning and controllable conversion of MoSe_2_ to MoS_2_, arrays of lateral MoSe_2_/MoS_2_ heterojunctions have been synthesized within a monolayer 2D crystal. Electron beam lithography coupled with vacuum deposition of SiO_2_ masks and sulfur, two scalable techniques used in the semiconductor industry, were employed to form sharp (∼5 nm) heterojunctions comparable to those formed by heteroepitaxy, without the non-uniformities typically introduced in VTG processes. Pulsed laser vaporization provided a controllable and rapid method to explore the conversion dynamics through the digital delivery of precise quantities of the sulfur precursor. The patterning and selective conversion process demonstrated here for semiconductor heterojunctions appears to be a powerful technique that could be extended to form other metallic, insulating and semiconducting regions within 2D materials required for ultrathin electronics.

## Methods

### E-beam patterning process

Electron beam lithography (FEI DB-FIB with Raith pattern writing software) was used for MoSe_2_/MoS_2_ heterostructure device fabrication. First, a layer of PMMA 495A4 was spin-coated on top of the MoSe_2_ flakes, followed by a 180°C bake on a hot plate. After pattern writing and developing, 50 nm of SiO_2_ was deposited using electron beam evaporation to serve as conversion masks. For device fabrication, 5 nm of Ti and 30 nm of Au were deposited using electron beam evaporation to serve as electrodes on the MoSe_2_ flakes. After the MoSe_2_ was converted to MoS_2_, electron beam lithography was conducted again to place electrodes on the MoS_2_. Finally, lift-off using acetone/IPA was used to reveal well-defined electrodes on both the MoSe_2_ and converted MoS_2_ flakes.

### Conversion process

The conversion was performed in a vacuum (∼10^−5^ Torr) chamber where a forward directed laser-vaporized sulfur plume was generated and digitally delivered to the surface of the crystals^28^. The vaporization target was prepared by compressing sulfur powder (99%, Sigma-Aldrich) into a pellet, and the laser fluence was adjusted to about 0.5 J cm^−2^ (spot size of 2 × 5 mm and repetition rate of 0.5 Hz) to ensure the transfer of a fixed amount of sulfur onto the crystals for all of the experimental conditions. The conversion was conducted under various substrate temperatures and laser pulses to understand and optimize the process (Supplementary Fig. 2; Supplementary Note 2).

### Optical characterizations

Raman and PL mapping and spectroscopy were performed in a Renishaw inVia micro-Raman system using a 532-nm laser excitation source, 1,800 g mm^−2^ grating and a laser power of about 1–5 mW through × 50 and × 100 objective lenses at room temperature. Maps were obtained with step sizes ranging from 0.3 to 1 μm. WiRE software was used to analyse and plot the maps for individual MoSe_2_/MoS_2_ sections and their overlaid images.

### STEM Z-contrast imaging and analysis

All STEM samples were baked at 160 °C for 8 h under vacuum before the microscopy experiment. STEM imaging was performed on an aberration-corrected Nion UltraSTEM-100 operating at 60 kV. The convergence semi-angle for the incident probe was 31 mrad. Z-contrast images were gathered for a half-angle range of ∼86–200 mrad. Electron energy loss spectroscopy and spectrum imaging was performed using a Gatan Enfina with a nominal collection angle of 35 mrad.

### Electrical transport characterization

The electrical measurement of MoSe_2_/MoS_2_ field-effect transistor devices was conducted in a vacuum chamber (∼10^−6^ Torr) using a Keithley 4200 semiconductor analyzer. The drain/source voltage (*V*_ds_) was set at 2 V with a gate voltage that swept from −60 to +60 V for transport measurement.

## Additional information

**How to cite this article:** Mahjouri-Samani, M. *et al.* Patterned arrays of lateral heterojunctions within monolayer two-dimensional semiconductors. *Nat. Commun.* 6:7749 doi: 10.1038/ncomms8749 (2015).

## Supplementary Material

Supplementary Information Supplementary Figures 1-5 and Supplementary Notes 1-5
