# Supplementary material for: Patterned arrays of lateral heterojunctions within monolayer two-dimensional semiconductors
Source: Nat Commun. 2015 Jul 22;6:7749. doi: 10.1038/ncomms8749 (PMC4525195; doi:10.1038/ncomms8749)
Supplement: Supplementary Information — Supplementary Figures 1-5 and Supplementary Notes 1-5 [file ncomms8749-s1.pdf]

Supplementary Figures

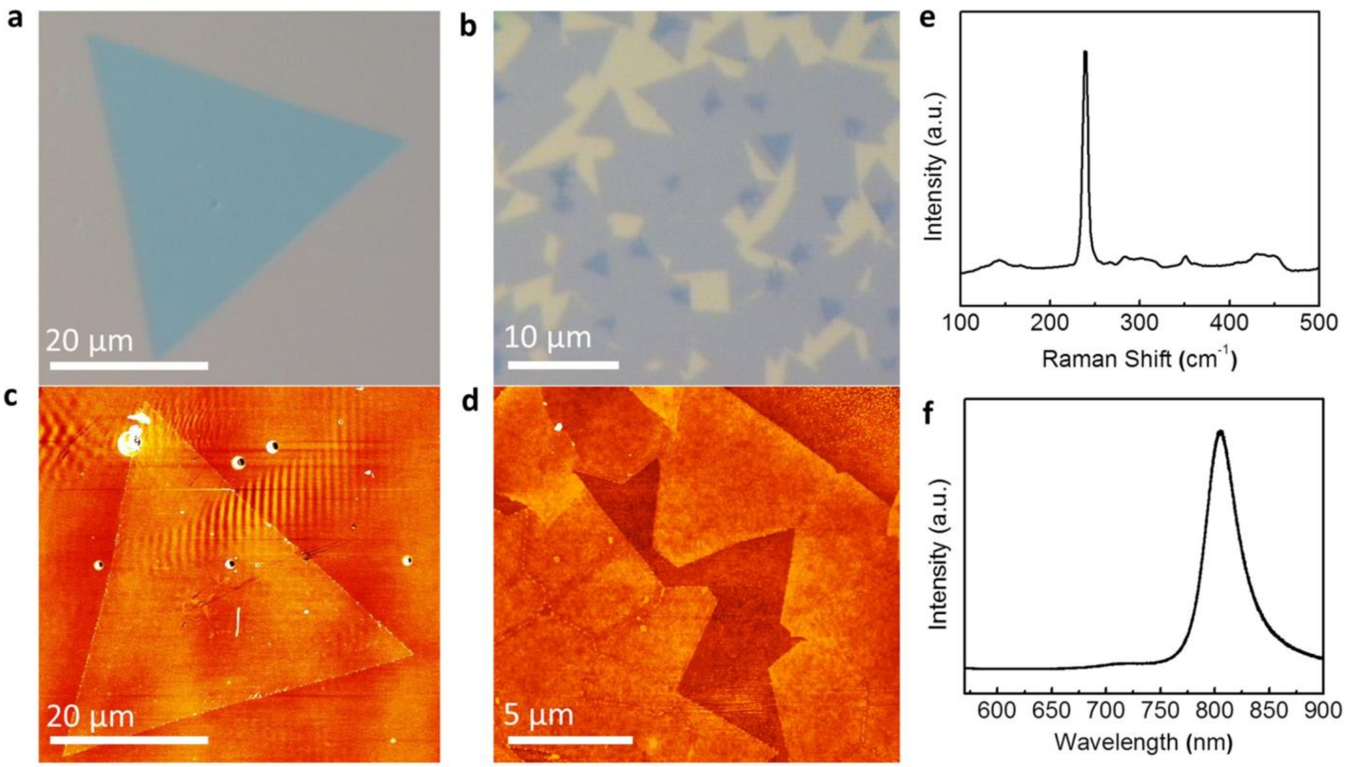

Supplementary Figure 1. $MoSe_2$ nanosheets synthesis and characterization. a-d, Optical and AFM images of typical isolated and merged monolayer $MoSe_2$ nanosheets grown in this work. **e, f,** Corresponding Raman and PL spectra indicating the E^1_{2g} Raman mode of $MoSe_2$ at 238 cm^{-1} and a PL peak at 805 nm.

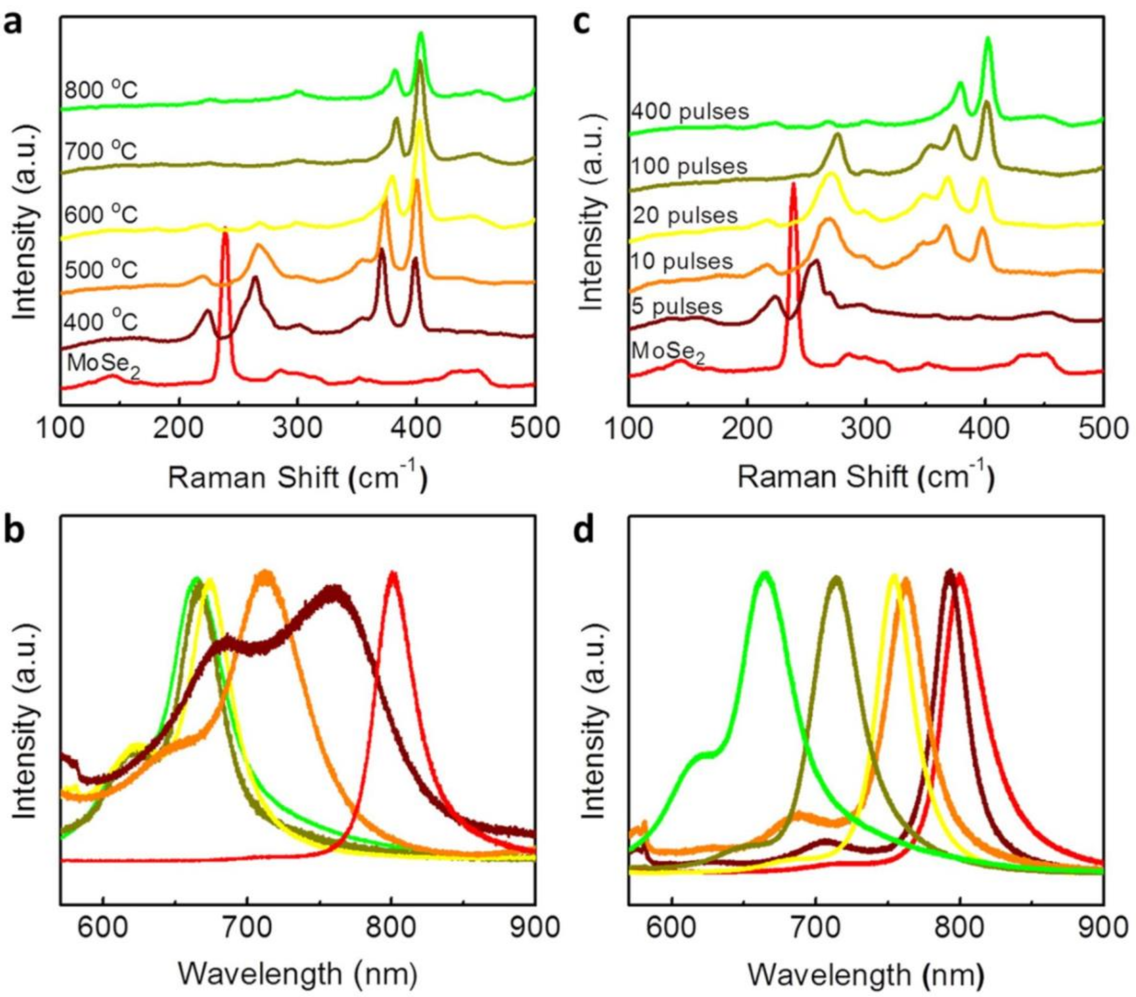

Supplementary Figure 2. Raman and photoluminescence characterization of various conversion conditions. a, b, Raman and corresponding PL spectra of a monolayer crystal under different conversion temperatures while keeping the other experimental conditions constant (i.e. 400 sulfur pulses, target to substrate distance of 11 cm, and laser fluence of 0.5 J/cm^2). At 400-500°C, the original Raman peak of the $MoSe_2$ nanosheet at 238 cm^{-1} disappears and MoS_2 peaks at 387 and 403 cm^{-1} appear. Also, the corresponding PL peak at 805 nm shifts to lower wavelengths. Disorder peaks at ~220 and 260 cm^{-1} indicate incomplete conversion and formation of intermediate alloys. Complete conversion is observed for temperatures above 600°C. **c, d,** Raman and PL spectra showing conversion evolution as a function of sulfur pulse number at a constant temperature (700°C) and the same distance (11 cm) and laser fluence (0.5 J/cm^2). The Raman and PL peaks of $MoSe_2$ slowly shift toward those associated with MoS_2 as the number of sulfur pulses increase, thereby resulting in a tunable bandgap.

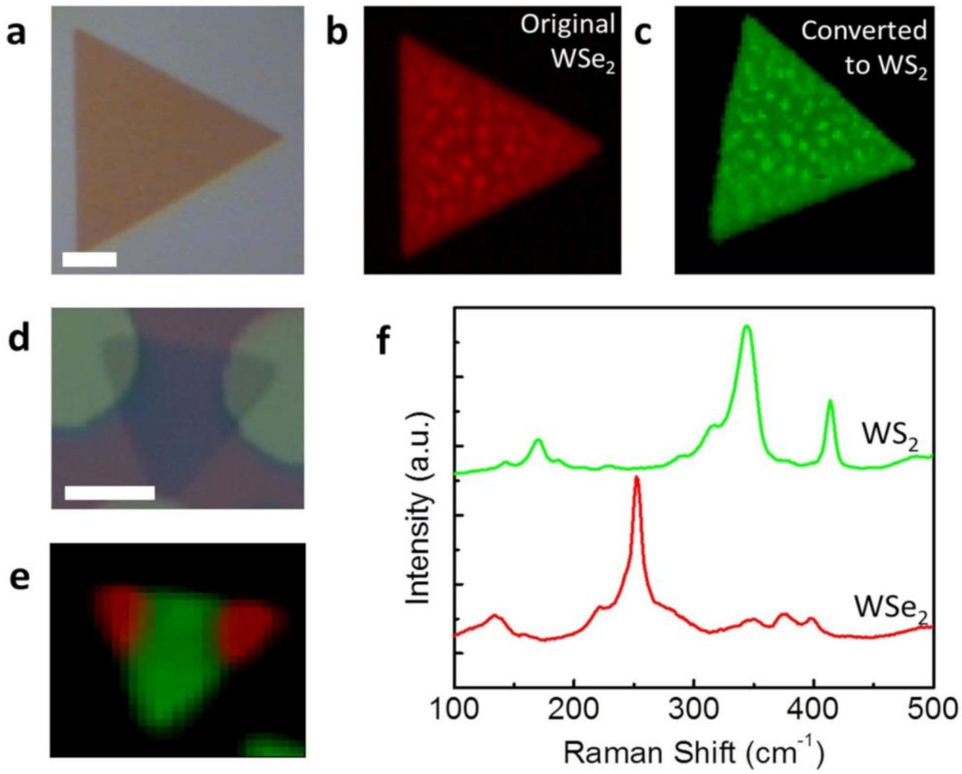

Supplementary Figure 3. Conversion of WSe_2 to WS_2 and formation of lateral heterojunctions. **a,** Optical image and **b,c,** Raman maps of a monolayer nanosheet before and after the complete conversion process (300 pulses at 700°C), respectively, indicating uniform of the crystal. **d, e,** Heterojunctions formed within monolayer crystals by patterning and selective conversion processes. The green and red Raman maps are obtained from corresponding optical images representing the WS_2 (intensity map at 350 cm^{-1}), WSe_2 (intensity map at 248 cm^{-1}) regions. **f,** Representative Raman spectra of the pristine WSe_2 and converted WS_2 regions. All of the scale bars are 5 μm in length.

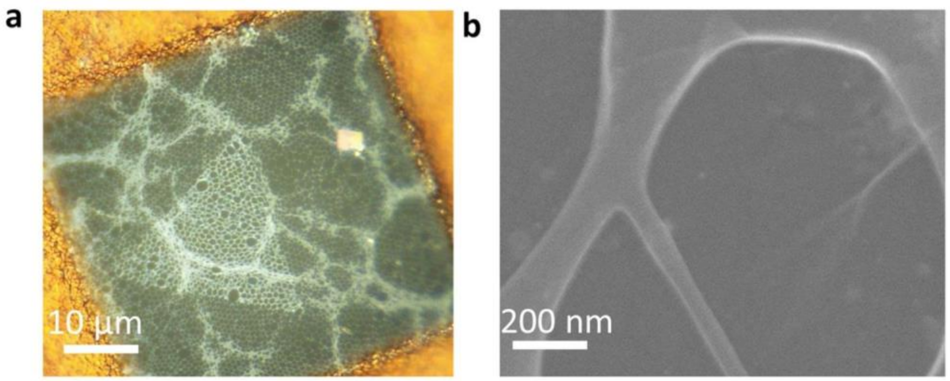

Supplementary Figure 4. TEM sample transfer. a, Optical image of a converted triangular crystal transferred onto a lacey carbon TEM grid. **b,** Magnified SEM image of a portion of the crystal suspended on the lacey carbon support.

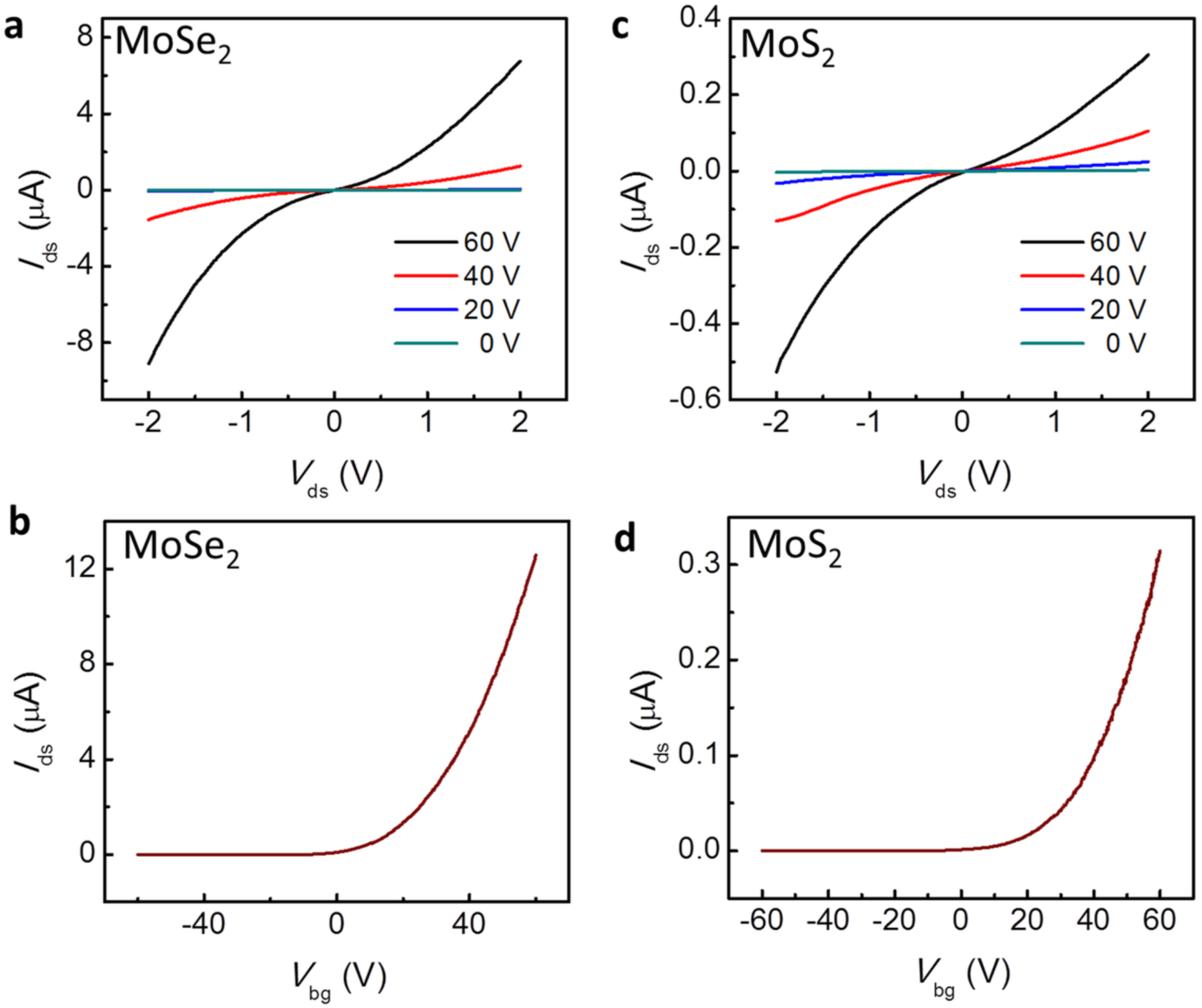

Supplementary Figure 5. Electrical transport characteristics were obtained from both pristine $MoSe_2$ and converted MoS_2 regions of the crystals. a, b, I_{ds}-V_{ds} and I_{ds}-V_{bg} of $MoSe_2$ region showing symmetrical curves with n-type transport characteristics. **c, d,** I_{ds}-V_{ds} and I_{ds}-V_{bg} of MoS_2 region showing symmetrical curves with n-type transport characteristics.

Supplementary Notes

Supplementary Note 1. Synthesis of $MoSe_2$ monolayer crystals

The $MoSe_2$ nanosheets were synthesized by PLD-assisted and conventional vapor phase transport (VTG) techniques. Conventional VTG involved vaporization of MoO_3 (99%, Sigma Aldrich) powder and selenium shots (99.99999%, Sigma Aldrich) in a tube furnace under argon (100 sccm) and hydrogen (10 sccm) flow and background pressure of 30 Torr at 800 ^{o}C for 30 min. PLD-assisted VTG, on the other hand, employed PLD to first deposit a uniform and precise amount of stoichiometric precursor nanoparticles onto a source substrate at room temperature that was then covered by a receiver substrate which was placed in contact and on top of the source substrate to form a confined VTG system. Both methods provided isolated and continuous monolayer $MoSe_2$ nanosheets. The as-synthesized $MoSe_2$ nanosheets were first identified by optical and atomic force microscopy (AFM) imaging and further studied under PL and Raman spectroscopy before patterning and conversion processes. As shown by optical and AFM images in Supplementary Figure 1a-d, both isolated and merged $MoSe_2$ monolayer crystals were obtained and identified. Supplementary Figure 1e, f show the corresponding Raman and PL spectra indicating the E^1_{2g} Raman mode of $MoSe_2$ at 238 cm^{-1} and a PL peak at 805 nm.

Supplementary Note 2. Conversion Process

Prior to the patterning process and formation of heterojunction arrays, the conversion of 2D monolayers was studied at various temperatures and laser pulse numbers to understand and optimize the process. PL and Raman spectroscopies were utilized to investigate the extent of conversion and to reveal their optical properties.

To investigate the effect of substrate temperature on the conversion process, a large number of laser pulses (400) were delivered to provide sufficient sulfur for reaction at different substrate temperatures (400-800°C). As shown by the Raman and PL spectra in Supplementary Figure 2a, b, $MoSe_2$ nanosheets were totally converted to MoS_2 for temperatures above 600°C. Below 600°C, however, intermediate

alloys where formed. At 400°C, for example, the E^1_{2g} mode of $MoSe_2$ at 238 cm^{-1} disappeared while the A^1_g and E^1_{2g} modes of MoS_2 at 383 and 403 cm^{-1} were observed. However, as can be seen from the spectra, the Raman peak at 250 cm^{-1} changes significantly, possibly due to the crystal lattice distortion by partial alloying. This peak slowly weakened as the temperature increased and completely disappeared at 700 and 800°C. The corresponding PL spectra also show the optical bandgap evolution at these temperatures. The effect of various laser pulse numbers was also investigated at a fixed substrate temperature. Supplementary Figure 2c, d shows the Raman and PL evolution as a function of laser pulse number at a constant substrate temperature of 700°C. Here, different laser pulse numbers ranging from 5 to 400 provided precise amounts of sulfur that resulted in a tunable bandgap that ranged from $MoSe_2$ at 800 nm to MoS_2 at 660 nm.

Supplementary Note 3. Conversion and formation of WSe_2/WS_2 heterojunctions

To show the broad and compelling technological advantages of our approach, we have also demonstrated the conversion of WSe_2 to WS_2 similar to $MoSe_2$/ MoS_2 process. We found that WSe_2 nanosheets are totally converted to WS_2 for substrate temperatures of 700°C and 300 laser-vaporized sulfur pulses. Supplementary Figure 3a-c shows the optical image and Raman maps of a WSe_2 monolayer before and after the conversion process. The WSe_2 and WS_2 Raman maps are plotted for the E^1_{2g} mode of WSe_2 at 248 cm^{-1} and E^1_{2g} modes of WS_2 at 350 cm^{-1}, respectively. Supplementary Figure 3d-e shows the optical image and Raman map of a patterned converted flake indicating the formation of heterojunction between the WSe_2 and WS_2 crystals. Representative Raman spectra of the flake before and after the conversion process are shown in Supplementary Figure 3f indicating WSe_2 and WS_2 Raman peaks similar to those reported in the literatures.

Supplementary Note 4. Sample transfer to TEM grids

The transfer of nanosheets onto TEM grids was performed by mechanical transfer using a PMMA-assisted method (PMMA=poly (methyl methacrylate) provided by Micro Chem, product number 950 PMMA A4). After spin-coating the sample with PMMA, the SiO_2 layer between the monolayer flakes

and the Si substrate was etched by a 2M KOH solution, thereby promoting lifted-off of the PMMA/flakes ensemble, which was then transferred onto TEM grids and allowed to air dry. The PMMA was then removed by soaking in acetone followed by a final rinse in isopropanol. Supplementary Figure 4a, b shows the optical and SEM images of a monolayer crystal transferred to a TEM grid.

Supplementary Note 5. First-principles calculations

First-principles calculations, based on density functional theory, were performed using the Vienna Ab initio Simulation Package. The projector augmented wave method was used to mimic the ionic cores, while the LDA considering the Ceperly–Alder–Perdew and Zunger (CA-PZ) functional was employed for the XC functional. Atomic positions, as well as lattice parameters, were optimized using a conjugate gradient algorithm. The ionic and electronic relaxations were performed by applying a convergence criterion of 1×10^{-2} eV/Å per ion and 10^{-5} eV per electronic step, respectively. The rectangular MoS_2–$MoSe_2$ hybrid structure, 44.1×3.2 Å, was used for the LDOS calculations, and a vacuum of 20 Å between the hybrids was considered. Also, 1×10×1 Monkhorst–Pack meshes were used to perform the integration over the Brillouin zone.
